# Inhibition of Drp1–Filamin Protein Complex Prevents Hepatic Lipid Droplet Accumulation by Increasing Mitochondria–Lipid Droplet Contact

**DOI:** 10.3390/ijms25105446

**Published:** 2024-05-17

**Authors:** Kohei Ariyoshi, Kazuhiro Nishiyama, Yuri Kato, Xinya Mi, Tomoya Ito, Yasu-Taka Azuma, Akiyuki Nishimura, Motohiro Nishida

**Affiliations:** 1 Department of Physiology, Graduate School of Pharmaceutical Sciences, Kyushu University, Fukuoka 812-8582, Japan; ariyoshi.kohei.438@s.kyushu-u.ac.jp (K.A.); knishiyama@omu.ac.jp (K.N.); yu-kato@phar.kyushu-u.ac.jp (Y.K.); mixinya@phar.kyushu-u.ac.jp (X.M.); ito.tomoya.214@m.kyushu-u.ac.jp (T.I.); 2 Laboratory of Prophylactic Pharmacology, Osaka Metropolitan University Graduate School of Veterinary Science, Osaka 598-8531, Japan; yta-vet@omu.ac.jp; 3 National Institute for Physiological Sciences (NIPS), National Institutes of Natural Sciences (NINS), Okazaki 444-8787, Japan; aki@nips.ac.jp; 4 Exploratory Research Center on Life and Living Systems (ExCELLS), National Institutes of Natural Sciences (NINS), Okazaki 444-8787, Japan; 5 Department of Physiological Sciences, School of Life Science, The Graduate University for Advanced Studies (SOKENDAI), Okazaki 444-8787, Japan

**Keywords:** lipid droplet, mitochondria fission, fatty liver disease, cilnidipine

## Abstract

Lipid droplet (LD) accumulation in hepatocytes is one of the major symptoms associated with fatty liver disease. Mitochondria play a key role in catabolizing fatty acids for energy production through β-oxidation. The interplay between mitochondria and LD assumes a crucial role in lipid metabolism, while it is obscure how mitochondrial morphology affects systemic lipid metabolism in the liver. We previously reported that cilnidipine, an already existing anti-hypertensive drug, can prevent pathological mitochondrial fission by inhibiting protein–protein interaction between dynamin-related protein 1 (Drp1) and filamin, an actin-binding protein. Here, we found that cilnidipine and its new dihydropyridine (DHP) derivative, 1,4-DHP, which lacks Ca^2+^ channel-blocking action of cilnidipine, prevent the palmitic acid-induced Drp1–filamin interaction, LD accumulation and cytotoxicity of human hepatic HepG2 cells. Cilnidipine and 1,4-DHP also suppressed the LD accumulation accompanied by reducing mitochondrial contact with LD in obese model and high-fat diet-fed mouse livers. These results propose that targeting the Drp1–filamin interaction become a new strategy for the prevention or treatment of fatty liver disease.

## 1. Introduction

Lipid droplets (LDs) represent dynamic and metabolically active cellular components, characterized by a hydrophobic core containing neutral lipids (mainly triglycerides and cholesterol esters) surrounded by a phospholipid monolayer [1,2]. This monolayer incorporates diverse proteins and enzymes involved in neutral lipid synthesis or metabolism. The liver is responsible for systemic lipid synthesis and metabolism, and the accumulation of LDs is a major symptom of fatty liver disease including nonalcoholic fatty liver disease (NAFLD) and nonalcoholic steatohepatitis (NASH). The number of patients diagnosed with NAFLD or NASH is increasing every year, and more effective therapies for NASH are needed. Liver biopsy is a diagnostic method for NASH, but it is burdensome and difficult to remark on as an early diagnostic method [3]. Hence, the establishment of early diagnosis and preventive treatment methods for this disease is urgently needed. Given this current situation, the development of drugs that inhibit LD accumulation in the liver is expected to be a new strategy to control the progression of fatty liver disease.

It has been suggested that mitochondria-mediated energy metabolism is associated with the formation and maintenance of LDs, and physical interactions between mitochondria and LDs are also implicated in the LD maintenance. Mitochondria intake fatty acids (FAs) and contribute to ATP production through the citric acid circuit and β oxidation [4]. Mitochondrial FA β oxidation stands as the primary pathway for FA degradation into acetyl units [5]. Under physiological conditions, the association of mitochondria with LDs is important in both lipid synthesis and metabolism aspects [4,6]. Mitochondria around the LD perform lipid synthesis via perilipin 5 and other factors [6]. Mitochondria are known to have high metabolic capacity via the citric acid circuit and electron transfer system [4]. Under cold conditions, brown adipocyte mitochondria actively induce uncoupling protein 1-mediated heat production by coming into contact with LDs [7]. In pathological conditions, however, dysfunctional mitochondrial bodies and metabolic abnormalities occur [8]. As a result, lipotoxicity is caused by the oxidation of free FAs and abnormal lipid metabolism [8,9]. The interplay between mitochondria and LDs assumes a crucial role in lipid metabolism [8]. Although mitochondria are dynamic organelles that can flexibly change their shape through fission and fusion [10], the relationship between mitochondrial quality and LDs is not well understood. Recent findings indicate that mitochondrial fusion is essential for oxidizing FAs released from LDs in mouse embryonic fibroblasts [11]. Dynamin-related protein 1 (Drp1) is activated locally at the site of mitochondrial fission through its interaction with actin [12]. We previously reported that the actin-binding protein filamin A (FLNA) acts as a guanine nucleotide exchange factor for Drp1 and mediates mitochondrial fission-associated myocardial senescence in mouse hearts after myocardial infarction [13,14]. In addition, cilnidipine, a dihydropyridine (DHP)-derived voltage-dependent L/N-type Ca^2+^ channel blocker that has been clinically approved as an anti-hypertensive drug, was found to inhibit the Drp1–FLNA interaction caused by hypoxic stress and myocardial senescence-associated chronic heart failure [13,14]. In this study, we seek to understand whether the Drp1–FLNA protein complex is involved in hepatic LD accumulation caused by a high-fat diet (HFD) and whether cilnidipine and its derivative, 1,4-DHP, which only lacks Ca^2+^ channel-blocking action of cilnidipine, has therapeutic potency with respect to fatty liver in obese mice.

## 2. Results

### 2.1. Treatment with Cilnidipine Reduced LDs Accompanied with Suppression of Palmitic Acid (PA)-Induced Drp1–FLNA Complex

**Figure 1:**
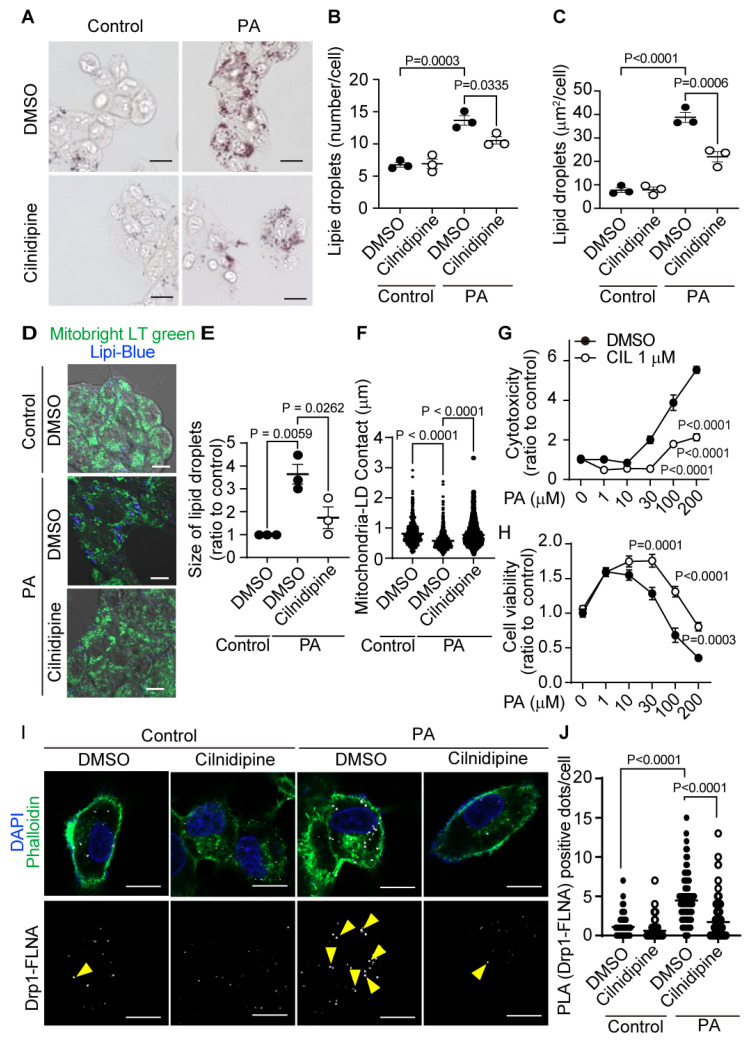
Treatment with cilnidipine reduces LDs in HepG2. (**A**–**C**) Effects of cilnidipine on palmitic acid (PA)-induced LDs. (**A**) Representative imaging of HepG2 cells treated with 30 μM of PA with or without cilnidipine (1 μM). (**B**,**C**) Number (**B**) and area (**C**) of LDs for each cell. (**D**) Representative images of contact between mitochondria (green) and LDs (blue). (**E**,**F**) Quantitative results of (**D**). (**E**) Size of LDs in each cell (n = 57–108 cells) and (**F**) area of mitochondria–LD contact (area shown by light blue). (**G**,**H**) Effects of cilnidipine on PA-induced (**G**) cytotoxicity and (**H**) viability. Cell death was induced by exposure to PA for 24 h. (**I**) Representative images of the Duolink proximity ligation assay (PLA) between Drp1 and FLNA. PLA signals are shown as white spots (yellow arrowhead) counterstained with phalloidin (green) and DAPI (blue). (**J**) Number of PLA signals for each cell with or without cilnidipine treatment (>150 cells). Data are means ± SEM (n = 3 in each group). Significance was determined using one-way ANOVA followed by Tukey’s comparison test. Scale bars: 10 μm (**A**,**D**,**I**).

We examined the effect of cilnidipine on PA-induced formation of LDs in HepG2 cells. Previous reports showed that the IC_50_ value for cilnidipine’s inhibition of mitochondrial hyperfission is approximately 70 nM, with a level of inhibition comparable to its L/N-type Ca^2+^ channel-blocking activity, and 1 μM cilnidipine can inhibit hypoxia-induced mitochondrial fission by >90%, without any toxicities in neonatal rat cardiomyocytes [13,15]. Treatment with 1 μM cilnidipine suppressed PA-induced increase in the size and number of LDs (Figure 1A–C). PA reduced the area of mitochondria–LD contact, and cilnidipine prevented the decrease in mitochondria–LD contact and cell death caused by PA exposure (Figure 1D–H). Treatment with cilnidipine suppressed PA-induced formation of the Drp1–FLNA protein complex (Figure 1I,J, Supplemental Figure S1). These data suggest that cilnidipine reduces PA-induced LD accumulation and cytotoxicity of HepG2 cells by preserving mitochondria–LD contact through inhibiting Drp1–FLNA interactions.

### 2.2. Treatment with Cilnidipine Improved Liver Injury and LDs in ob/ob Mice

**Figure 2:**
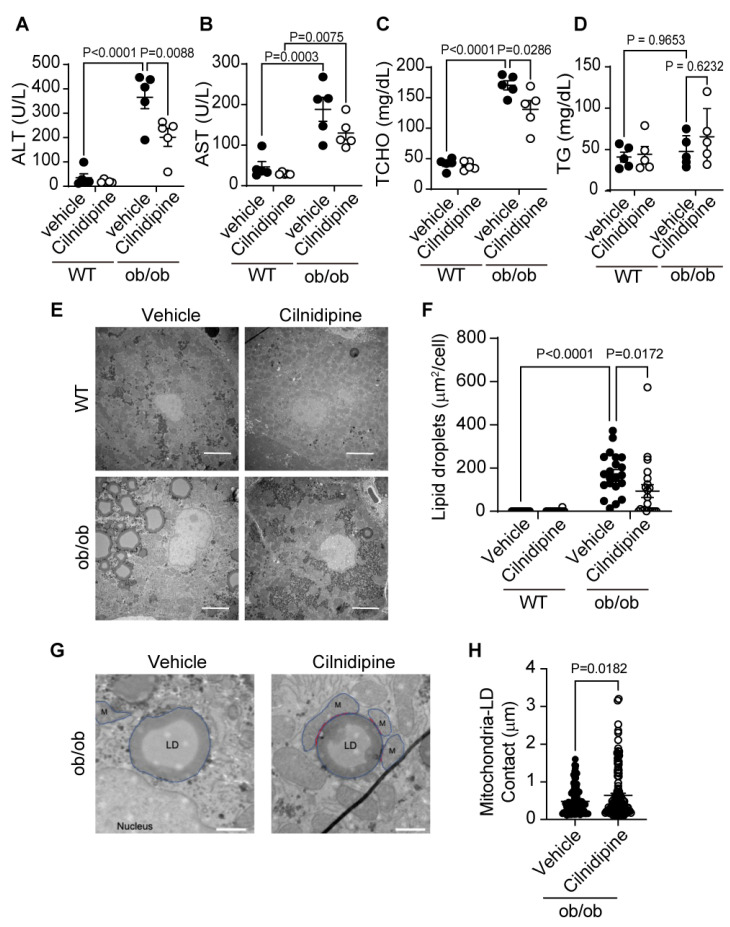
Treatment with cilnidipine improves liver injury and LDs in ob/ob mice. (**A**–**D**) Effect of cilnidipine on plasma levels of ALT (**A**), AST (**B**), TCHO (**C**) and TG (**D**) (n = 5 mice in each group). (**E**) Representative TEM images of mouse livers. (**F**) Quantitative result of LD areas using ImageJ (Version 1.54g) n = 30 cells in each group). (**G**) Representative TEM images of mitochondria–LD contact (shown by red line). Individual mitochondria and LD are marked in blue line. (**H**) Quantitative result of mitochondria–LD contact using ImageJ (n = 30 cells in each group). Data are means ± SEM. Significance was determined using one-way ANOVA followed by Tukey’s comparison test. Scale bars: 5 μm (**E**) and 1 μm (**G**).

Next, we investigated whether the treatment of cilnidipine suppresses the progression of NAFL using two hyperlipidemic NAFL model mice. Liver damages were assessed by plasma levels of alanine aminotransferase (ALT) and aspartate aminotransferase (AST). Treatment with cilnidipine attenuated the increases in plasma ALT and AST levels of ob/ob mice (Figure 2A,B). Cilnidipine also suppressed the increase in plasma total cholesterol (TCHO) level, an index for lipid metabolism (Figure 2C). Triglyceride (TG) level was not increased in ob/ob mice (Figure 2D). Transmission electron microscope (TEM) images revealed the obvious induction of steatosis in ob/ob mouse livers (Figure 2E). The average volume of LDs was significantly increased in ob/ob mice compared with wild-type (WT) mice. Treatment with cilnidipine decreased LD volume (Figure 2E,F), and increased contact between mitochondria and LDs (Figure 2G,H). The physical contact between mitochondria and LDs are reportedly implicated in LD synthesis and lipid degradation/lipid metabolism [4,6]. Not only mitochondrial fission factors but also fusion factors, endoplasmic reticulum (ER) stress, oxidative stress, inflammation and others are known to cause abnormalities in lipid metabolism [16,17]. However, focusing on mRNA expression changes, the expression levels of genes related to mitochondrial fusion factors (Opa1, mitofusin (Mfn) 1/2), ER stress (ATF6) and inflammation (IL-6, TNF-α, IL-1β) were not significantly increased in the liver of ob/ob mice (Supplemental Figure S2). These data suggest that cilnidipine improves hyperlipidemia in the liver by increasing mitochondria–LD contact.

### 2.3. Treatment with Cilnidipine Improved HFD-Induced LDs

**Figure 3:**
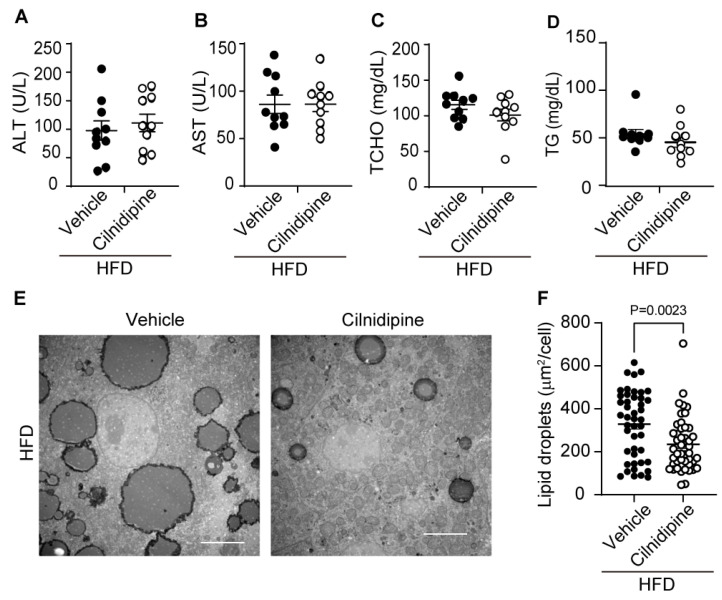
Treatment with cilnidipine improves HFD-induced LD accumulation. (**A**–**D**) Effect of cilnidipine on plasma levels of ALT (**A**), AST (**B**) TCHO (**C**) and TG (**D**) (n = 10 mice in each group). (**E**) Representative TEM images of livers. Scale bar: 5 μm. (**F**) Quantitative result of LD formation using ImageJ (n = 30 cells in each group). Data are means ± SEM. Significance was determined using student’s *t*-test.

In mice with HFD, the increases in plasma ALT, AST, TCHO, and TG levels were mild compared with data ob/ob mice (Figure 2A–D) and cilnidipine failed to reduce them (Figure 3A–D). However, cilnidipine treatment significantly decreased liver LD volumes in mice fed with HFD (Figure 3E,F). In the livers of mice fed HFD, the expression levels of mRNAs related to mitochondrial fusion factors, inflammatory factors, ER stress-related factors, as well as oxidative stress (SOD1 and SOD2) and autophagy (PINK), were not significantly changed (Supplemental Figure S3). These data suggest that cilnidipine can suppress HFD-induced LD accumulation in the liver, despite insufficient therapeutic effects for mild liver impairment.

### 2.4. Treatment with 1,4-DHP Also Reduced LDs and Suppressed PA-Induced Drp1–FLNA Complex

**Figure 4:**
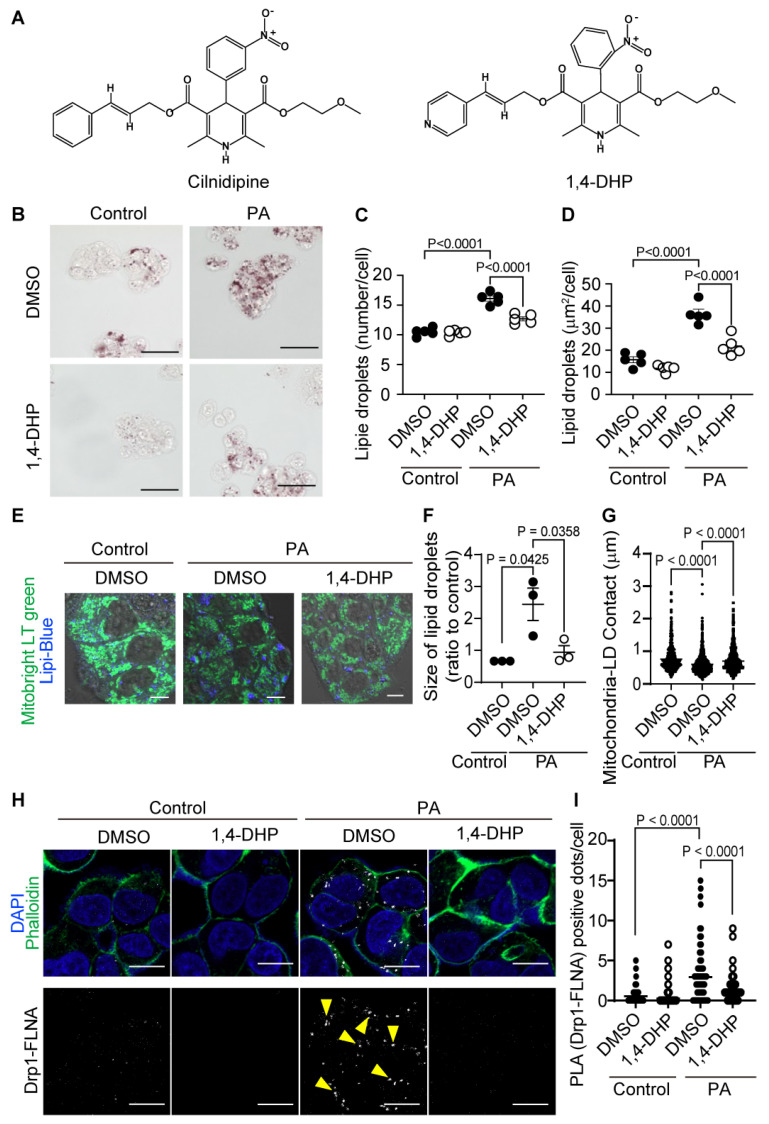
1,4-DHP reduces PA-induced LD accumulation in HepG2. (**A**) Chemical structures of cilnidipine and 1,4-DHP. (**B**–**D**) Effects of 1,4-DHP on PA-induced LDs. (**B**) Representative imaging of HepG2 cells treated with 30 μM of PA with or without 1,4-DHP in HepG2. Number (**C**) and area (**D**) of LDs for each cell. (**E**) Representative images of contact between mitochondria (green) and LDs (blue). (**F**,**G**) Quantitative results of (**E**). (**F**) Size of LDs in each cell (n = 63–108 cells) and (**G**) the area of mitochondria–LD contact (shown by merged color (light blue)). (**H**) Representative PLA images between Drp1 and FLNA. PLA signals are shown as white spots (yellow arrowhead) counterstained with phalloidin (green) and DAPI (blue). (**I**) Number of PLA signals for each cell with or without 1,4-DHP treatment (>150 cells). Data are means ± SEM (n = 3–5 in each group). Significance was determined using one-way ANOVA followed by Tukey’s comparison test. Scale bars: 10 μm (**B**,**E**,**H**).

Cilnidipine has Ca^2+^ channel inhibitory activity and decreases blood pressure. We recently developed a dihydropyridine derivative without Ca^2+^ channel inhibitory activity, (E)-3-(2-methoxyethyl) 5-(3-(pyridin-4-yl)allyl) 2,6-dimethyl-4-(2-nitrophenyl)-1,4-dihydropyridine-3,5-dicarboxylate (1,4-DHP), which suppressed mitochondrial fission induced by hypoxia without Ca^2+^ channel inhibitory activity (Figure 4A) (WO2020241638 (Akio Ojida, Naoya Shindo, Akiyuki Nishimura, Motohiro Nishida)). Treatment of HepG2 cells with 1,4-DHP suppressed PA-induced formation of LDs (Figure 4B–D). Treatment with 1,4-DHP suppressed the reduction of mitochondria–LD contact caused by PA exposure (Figure 4E–G). Additionally, 1,4-DHP suppressed PA-induced Drp1–FLNA protein complex formation (Figure 4H,I, Supplemental Figure S4). These data suggest that 1,4-DHP reduces LD accumulation through inhibiting Drp1–FLNA interactions.

### 2.5. Treatment with 1,4-DHP Improved Liver Injury and LDs in ob/ob Mice Fed HFD

**Figure 5:**
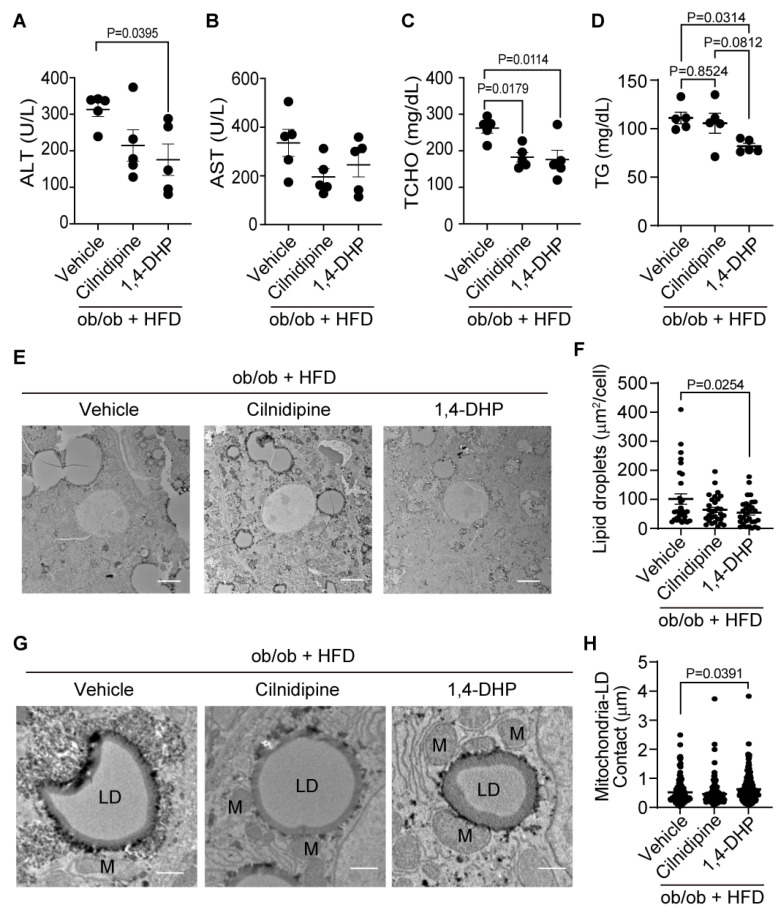
Treatment with 1,4-DHP improves liver injury and LDs in ob/ob mice fed HFD. (**A**–**D**) Effect of cilnidipine or 1,4-DHP on plasma levels of ALT (**A**), AST (**B**), TCHO (**C**) and TG (**D**) (n = 5 mice in each group). (**E**) Representative TEM images of livers. (**F**) Quantitative data of steatosis using ImageJ. (**G**) Representative TEM images of mitochondria–LD contact. (**H**) Quantitative data of mitochondria–LD contact using ImageJ (n = 30 cells in each group). Data are means ± SEM (n = 30 cells in each group). Significance was determined using one-way ANOVA followed by Tukey’s comparison test. Scale bars: 5 μm (**E**) and 1 μm (**G**).

We further investigated whether inhibition of the Drp1–FLNA complex could improve severe hepatic steatosis, using ob/ob mice fed with HFD. Treatment with cilnidipine failed to attenuate the increases in plasma ALT levels in ob/ob mice fed HFD, while treatment with 1,4-DHP significantly reduced plasma ALT level in ob/ob mice fed HFD (Figure 5A). Plasma AST was unchanged among respective groups (Figure 5B). Treatment with cilnidipine and 1,4-DHP prevented the increase of TCHO (Figure 5C), and 1,4-DHP significantly reduced plasma TG level (Figure 5D). Treatment with 1,4-DHP decreased LD accumulation in the liver of ob/ob mice fed HFD (Figure 5E,F). TEM images revealed that 1,4-DHP increased physical contact between mitochondria and LDs (Figure 5G,H). In the liver of the HFD-fed ob/ob mouse, there were no significant changes in mRNA expression levels related to mitochondrial fusion, inflammation, oxidative stress or ER stress (Supplemental Figure S5). These data suggest that selective inhibition of Drp1–FLNA interactions by 1,4-DHP improves dyslipidemia and LD accumulation in the liver by increasing mitochondria–LD contact.

### 2.6. Knockdown of Drp1 and FLNA Suppressed LD Formation in HepG2 Cells

**Figure 6:**
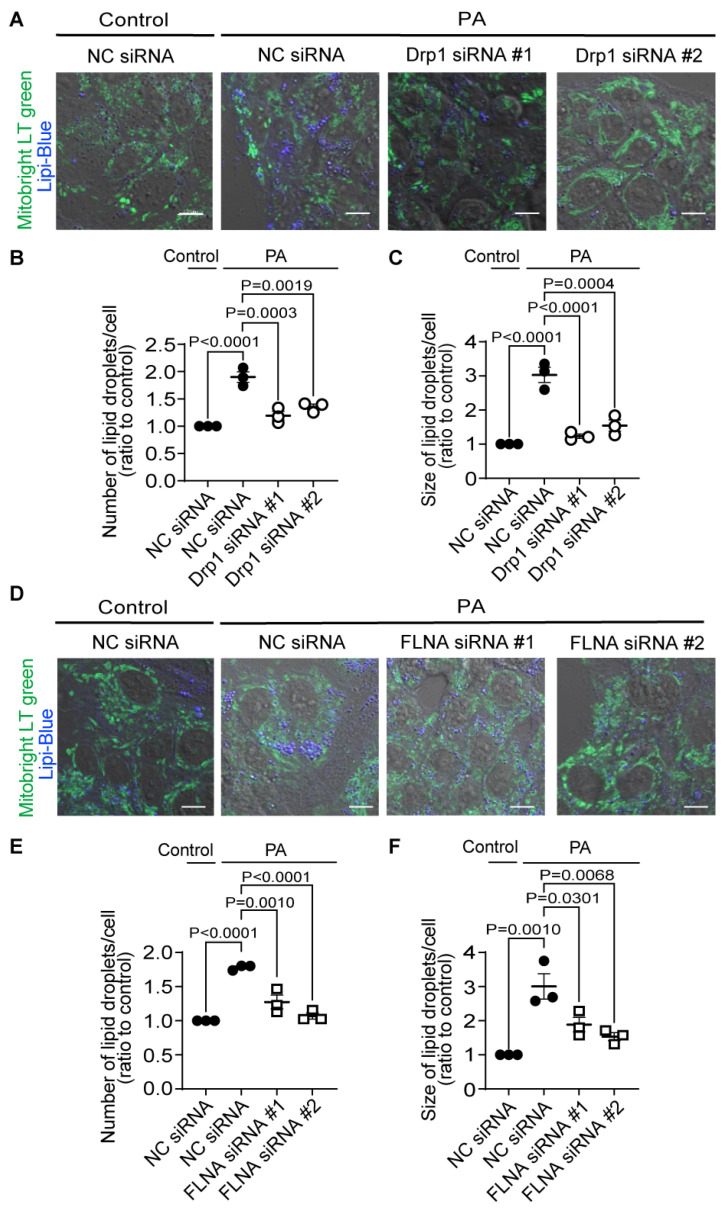
Knockdown of Drp1 and FLNA suppresses LD formation in HepG2 cells. (**A**–**C**) Effect of Drp1 siRNA knockdown on LD accumulation of HepG2 cells exposed to PA (30 μM) for 24 h. (**A**) Representative images of HepG2 cells treated with or without Drp1 siRNA. Average number (**B**) and area (**C**) of LDs in each cell (n = 65–115 cells per experiment). (**D**–**F**) Effect of FLNA siRNA on PA-induced LD formation. (**D**) Representative images of HepG2 cells treated with or without FLNA siRNA. Average number (**E**) and area (**F**) of LDs in each cell (n = 70–129 cells per experiment). NC: negative control. Data are means ± SEM (n = 3 in each group). Significance was determined using one-way ANOVA followed by Tukey’s comparison test. Scale bars: 10 μm (**A**,**D**).

We tested whether the Drp1–FLNA complex is directly involved in LD formation by silencing Drp1 and FLNA. We validated the knockdown efficiency of siRNAs, and confirmed that the mRNA expression levels of Drp1 and FLNA were markedly decreased by the treatment with siRNA (120 nM) (Supplemental Figure S6A,B). However, as the cellular conditions were not optimal under 120 nM treatment, we reduced the siRNA concentration to 30 nM, where we preliminarily confirmed that it exhibited a knockdown efficiency comparable to 120 nM without causing cell damage. Subsequently, upon treating HepG2 cells with each siRNA (30 nM), we found a significant inhibition of the increase in LD number and the enlargement of LDs caused by PA exposure (Figure 6A–D). While it cannot be concluded solely based on these results that cilnidipine and 1,4-DHP directly inhibits the Drp1–FLNA protein complex, considering the inhibitory effect of cilnidipine and 1,4-DHP on the Drp1–FLNA complex in the PLA assay and our previous reports using cardiac cells [13], cilnidipine and 1,4-DHP may suppress the PA-induced increase in LD number and the enlargement of LDs by inhibiting Drp1–FLNA complex formation.

### 2.7. Cilnidipine Improved Fatty Acid Oxidation (FAO)

**Figure 7:**
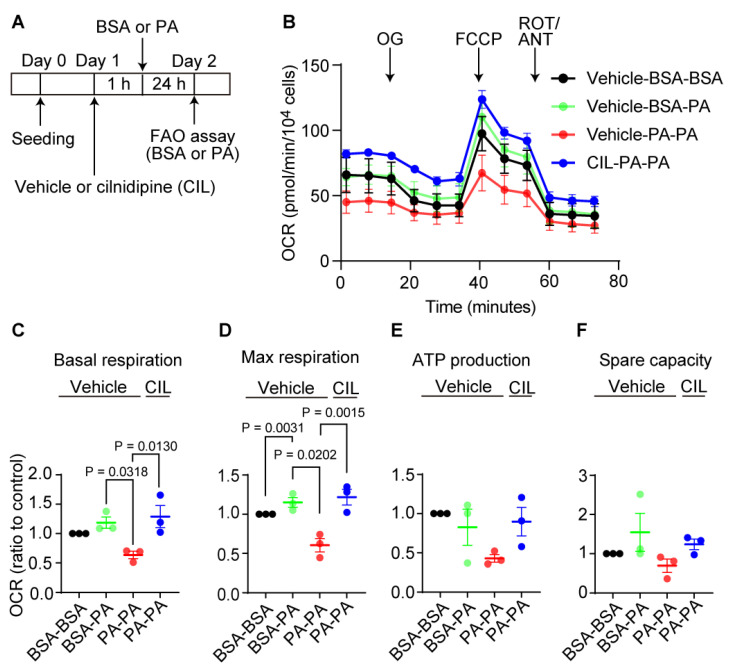
Cilnidipine improves PA-induced respiratory dysfunction of HepG2 cells. (**A**) Experimental scheme of FAO assay. (**B**) Oxygen consumption rate (OCR) of HepG2. Cells were pretreated with 200 μM PA or BSA for 24 h with or without 1 μM cilnidipine in substrate-limited D-MEM media supplemented; cells were changed in FAO assay medium and incubated in non-CO_2_ free incubator. Cells were stimulated with PA or BSA just before OCR measurement. Group names were defined by the order in which reagents were added. OL: oligomycin, FCCP: carbonyl cyanide *p*-(trifluoromethoxy) phenylhydrazone, ROT: Rotenone, ANT: antimycin. (**C**–**F**) Average basal respiration (**C**), maximal respiration (**D**), ATP production (**E**) and spare capacity (**F**). Data are means ± SEM (n = 3 in each group). Significance was determined using one-way ANOVA followed by Tukey’s comparison test.

Next, we tested whether cilnidipine prevents metabolic dysfunction caused by long-time PA exposure using FAO assay (Figure 7A) [18]. Exposure to excess fatty acid is reported to cause reduction of mitochondrial function in cells [19]. Treatment with PA for 24 h reduced the basal and maximal oxygen consumption rate (OCR) in HepG2 cells, and cilnidipine treatment prevented the PA-induced respiratory dysfunction (Figure 7B–D). ATP production and spare capacity were not significantly changed (Figure 7B,E,F). These data suggest the protective effect of cilnidipine against PA-induced abnormal fatty acid metabolism.

## 3. Discussion

During periods of fasting and feeding, hepatocytes engage in the synthesis, storage and secretion of lipids to uphold overall lipid homeostasis. The processes of external FA uptake and internal de novo synthesis are intricately balanced by the rates of FA degradation and the secretion of bile acids into canaliculi via predominantly ATP-dependent transporters, along with the release of lipoproteins into circulation through the secretory apparatus. These processes are subject to regulation by various factors, including substrate availability and metabolic hormones. Under normal physiological conditions, the liver stores only modest amounts of FAs as lipids in cytosolic LDs [20]. Disruptions in the equilibrium among LD formation, mobilization and the secretion of lipoproteins or bile acids can result in pathological lipid accumulation, a condition such as NAFLD. This often manifests in cases of obesity, where adipose tissue exceeds its lipid storage capacity, leading to an overflow of lipids into the liver [21]. Characterized by the formation of numerous large LDs in hepatocytes, NAFLD encompasses various histopathological stages, ranging from clinically asymptomatic hepatic steatosis to NASH, marked by inflammation, fibrosis and occasionally cirrhosis [1,22]. However, not all obese individuals develop steatosis, and only a small percentage progress to NASH [23,24]. The cellular processes underlying the development of NAFLD and progression to NASH remain inadequately understood. In the past decade, genome-wide association studies have identified multiple genomic sequence variations linked to an elevated risk of chronic liver disease [25,26]. Obesity and dietary factors significantly exacerbate this risk in individuals carrying these identified genetic factors [27]. As obesity becomes more prevalent, NAFLD is on the rise, affecting approximately 20–30% of the population in Western countries and a total of around 1.8 billion people worldwide [28,29,30]. A comprehensive understanding of the cellular and molecular processes disrupting liver lipid metabolism is crucial to identifying novel pharmacological targets and developing effective treatment strategies.

We used ob/ob mice and HFD-fed mice as a mild fatty liver model. Generally, in fatty liver, abnormal increases in serum parameters such as AST, ALT [23]. In this study, we showed that excessive fat accumulation occurs in ob/ob mice and HFD-fed mice. We also showed that AST and ALT were increased in ob/ob mice compared with WT mice. Therefore, these data suggest that the ob/ob mice and the HFD-fed mice mimic mild fatty liver disease. Treatment of cilnidipine reduced hepatic LDs in ob/ob mice and HFD-fed mice. In ob/ob mice, treatment of cilnidipine improved liver function markers such as AST and ALT. On the other hand, treatment of cilnidipine did not improve AST and ALT in mice fed HFD. Mitochondrial dysfunction has been identified in liver tissue of individuals with fatty liver disease, manifesting through varying degrees of ultrastructural damage to mitochondria, abnormal morphological alterations, reduction in respiratory chain activity, ATP depletion, heightened permeability of both outer and inner mitochondrial membranes, excessive reactive oxygen species (ROS) production, oxidative stress-mediated deletions of mitochondrial DNA and impaired mitochondrial β-oxidation [31,32,33]. Recent investigations strongly suggest that mitochondrial dysfunction plays a pivotal role in the onset and progression of NAFLD [34]. However, the precise mechanism by which mitochondrial dysfunction contributes to NAFLD remains not entirely elucidated. Factors such as diminished fatty acid oxidation (FAO), increased delivery and transport of free FAs into the liver, and elevated hepatic FA synthesis have been implicated in the pathogenesis of NAFLD [31]. Mitochondrial dysfunction is associated with a decrease in β-oxidation of lipids, leading to the accumulation of triglycerides within hepatocytes [35]. In individuals with obesity and NAFLD, hepatocyte electron transport chain function has been reported to moderately malfunction [36]. We also demonstrated that cilnidipine inhibited PA-induced Drp1–FLNA complex formation in an in vitro system. This suggests that improvement of mitochondrial function by inhibiting Drp1–FLNA complex formation alleviates the symptoms of fatty liver.

Recently, another research group has delved into the intricate relationship among NAFLD/NASH, mitochondrial FAO in the liver and hepatic mitochondrial quality in human subjects [37]. Liver biopsies were procured from obese patients undergoing bariatric surgery, and they systematically correlated liver histology with the measured mitochondrial FA oxidation in the liver tissue. The outcomes of this investigation unveiled a substantial reduction, approximately 40–50%, in hepatic mitochondrial FA β oxidation in subjects with NASH compared to control subjects exhibiting normal histology. This decline was accompanied by an elevation in hepatic mitochondrial ROS production and reductions in markers of mitochondrial biogenesis and mitophagy [37]. We have not fully investigated the effects of CIL and 1,4-DHP treatments on mitochondrial oxidative stress occurring in hepatocytes under hyperglycemic and hyperlipidemic conditions. In the future, conducting analyses using techniques such as Mitosox staining may further substantiate the hepatoprotective effects of CIL and 1,4-DHP. These findings provide compelling evidence supporting the association between mitochondrial dysfunction and NAFLD/NASH in humans, suggesting that impaired hepatic FAO and diminished mitochondrial quality are closely linked to the severity of NAFLD in individuals with obesity. We showed that treatment with cilnidipine increased the contact between mitochondria and LDs. Contact between mitochondria and LDs involves both LD synthesis and lipid degradation/lipid metabolism [38]. Perilipin 5-mediated mitochondria–LDs interaction promotes LD expansion [38]. The mitochondrial proteins that interact with perilipin 5 are still unknown. On the other hand, the interaction between perilipin 1 and Mfn2 is induced during lipolysis and increases mitochondria–LD contact [38]. The mechanism of interaction that controls the binding between mitochondria and LDs under hyper-nutrition remains unclear [38]. The liver harbors two kinds of mitochondria, cytoplasmic mitochondria and LD-associated mitochondria (LDM) [39]. It was reported that LDM is specialized and adapted for FAO. Hallmarks of liver LDM under an ad libitum condition include increased FAO, decreased tricarboxylic acid flux, decreased membrane potential, decreased complex I + III and II + III activities, decreased ATP levels, increased carnitine palmitoyl transferase 1 activity, elevated Mfn2 levels, inactivated acetyl-CoA carboxylase 2 and decreased LDs size [39]. In brown fat cells, the peroxisome proliferator-activated receptor gamma plays an important role in heat production [40]. In this study, we showed that cilnidipine inhibits Drp1–FLNA complex formation in HepG2 cells and reduces LD accumulation in hyperlipidemic mouse livers. As cilnidipine restored the PA-induced FAO reduction (Figure 7), cilnidipine may have contributed to the treatment of fatty liver disease through preserving FAO activity.

The Drp1–FLNA complex was formed during excessive FA supply or fatty liver. We previously reported that the formation of the Drp1–FLNA complex is promoted during hypoxia [13,14]. Accumulating evidence from the past few decades provides strong support for the existence of interruptions in oxygen availability in fatty livers [7]. Furthermore, activation of hypoxia-inducible factor 1-alpha is known to advance NAFLD [41,42]. Hypoxia response during fatty liver may promote the formation of Drp1–FLNA complex. It has been reported that hypoxic reactions are involved not only in ischemic diseases but also in various diseases such as amyotrophic lateral sclerosis and viral hepatitis [43]. Drp1–FLNA complex formation during hyperlipidemia may facilitate the impairment of FA metabolism through mitochondrial dysfunction, leading to NAFLD progression. Targeting the Drp1–FLNA complex may modulate mitochondrial morphology and control these hypoxia-related diseases.

Treatment of cilnidipine failed to suppress hepatic steatosis in HFD-fed ob/ob mice. On the other hand, treatment of 1,4-DHP suppressed fat accumulation in the liver and improved liver function (Figure 5). Cilnidipine is usually used as anti-hypertensive drug by L/N type Ca^2+^ channel blocker in clinical scenes, and it is possible that it could not improve more severe diseases such as decreased insulin secretion due to Ca^2+^ channel blockade. Since 1,4-DHP does not inhibit L/N-type Ca^2+^ channels, 1,4-DHP may have little adverse effect on insulin secretion ability. Thus, improving mitochondrial quality by inhibiting Drp1–FLNA complex formation without inhibiting Ca^2+^ signals will be a promising strategy for alleviating hepatic steatosis.

Ca^2+^ homeostasis is related to the endoplasmic reticulum and mitochondria. Abnormalities in intracellular Ca^2+^ are also associated with the onset of NAFLD/NASH [44]. 1,4-DHP does not inhibit Ca^2+^ channel activity. Since 1,4-DHP improved severe fatty liver disease, 1,4-DHP may also improve signal transduction between the endoplasmic reticulum and mitochondria. It is also possible that the formation of the Drp1–FLNA complex due to high-fat stimulation weakens the interaction between mitochondria and the endoplasmic reticulum, leading to a decline in mitochondrial function.

In this study, we showed that cilnidipine improved mild fatty liver disease. We showed that 1,4-DHP improves fat accumulation and liver function in severe fatty liver. Treatment of cilnidipine or 1,4-DHP increased contact between mitochondria and LDs. These data suggest that increasing mitochondria–LD interactions improves lipid metabolism. Furthermore, in severe fatty liver disease, the interaction between mitochondria, LDs and the endoplasmic reticulum may become important in the progression of the disease. On the other hand, it is also known that fatty liver and high blood pressure tend to coexist. When fatty liver and hypertension coexist, cilnidipine may be more beneficial for patients. This study suggests that elucidation of the interaction between mitochondria, endoplasmic reticulum and LDs and its regulatory mechanism through the Drp1–FLNA complex can lead to new therapeutic strategies for fatty liver disease such as NAFLD/NASH.

## 4. Materials and Methods

### 4.1. Cell Culture and Oil-Red-O Staining

We cultured HepG2 cells in low glucose (5 mM) Dulbecco’s modified eagle medium (DMEM) supplemented with 10% FBS and 1% penicillin and streptomycin. We seeded HepG2 cells at a density of 2 × 10^4^ cells/well on 35 mm Glass Bottom dish (MATSUNAMI, Osaka, Japan) and incubated cells overnight. Cells were treated with or without cilnidipine (1 μM) or 1,4-DHP (1 μM) for 1 h and then PA (30 μM) was added for 24 h. BSA conjugated PA (Cayman Chemical, Ann Arbor, MI, USA) was used for PA, and BSA solution (Cayman) of the same composition was used as control. For Control, BSA solution was diluted so that the final concentration of diluted BSA conjugated PA and BSA were the same. We assessed PA-induced LD formation in HepG2 cells by Oil-Red-O staining. After fixation in 4% paraformaldehyde (PFA) for 10 min, the cells were stained with Oil-red-O solution for 20 min. The Oil-Red-O stock solution was prepared by mixing 10 mL of 99% isopropanol with 30 mg of Oil-Red-O and heating the mixture at 60 °C overnight. For experimental use, the Oil-Red-O stock solution was combined with Milli-Q deionized water at a ratio of 6:4 to prepare the Oil-Red-O working solution. The Oil-Red-O working solution was used within 1 h after preparation. After replacing with Ca^2+^ and Mg^2+^-free phosphate buffered saline (PBS), the cell images were captured by BZ-800 microscopy (KEYENCE, Osaka, Japan).

### 4.2. Palmitate (PA)-Induced Cell Death

HepG2 cells were seeded at a density of 2 × 10^4^ cells/well on 96-well culture plates and incubated overnight. Cells were treated with or without cilnidipine (1 μM) for 1 h and then PA was added for 24 h. We assessed PA-induced cell death in HepG2 cells by measuring LDH release and MTT assays as described previously [45].

### 4.3. Live Cell Imaging

HepG2 cells with cilnidipine (1 μM) or 1,4-DHP (1 μM) and PA (30 μM) were washed with Ca^2+^ and Mg^2+^-free Hank’s Balanced Salt Solution (HBSS) (Thermo Fisher Scientific, Waltham, MA, USA). Then cells were stained with Lipi-blue (200 nM) and Mitobright LT Green (100 nM) (Dojindo, Kumamoto, Japan) and incubated at 37 °C. After staining, cells were washed with HBSS twice. Images were captured using a confocal microscope (LSM900, ZEISS, Oberkochen, Germany).

### 4.4. Visualization of Drp1–FLNA Interactions

To determine Drp1 and filamin interaction in HepG2, PLA assay was conducted using Duolink PLA Fluorescence (Sigma Aldrich, Burligton, MA, USA) according to the manufacturer’s instructions. HepG2 cells were seeded at a density of 1.5 × 10^4^ cells/well on flexiparm. Cells were treated with or without cilnidipine (1 μM) or 1,4-DHP (1 μM) for 1 h and then PA (30 μM) was added for 24 h. After 4% PFA fixing and blocking, cells were incubated with mouse anti-Filamin (Santa Cruz, Dallas, TX, USA, 1:50) and rabbit anti-Drp1 (Abcam, Cambridge, UK, 1:500) at 4 °C overnight followed by PLA probe incubation for 1 h. The ligation (1 h) and amplification (2 h) steps were performed in 37 °C chamber and livers were nuclear stained with DAPI and phalloidin. Images were captured using a confocal microscope (LSM900, ZEISS).

### 4.5. Animals

Male C57BL/6 mice (19–23 g, 7–10 weeks old) and ob/ob mice were obtained from SLC (Shizuoka, Japan). All mice were housed in individually ventilated cages with poplar wood chip bedding in groups of three mice per cage and maintained under controlled environmental conditions (12 h light/12 h dark cycle, room temperature 21–23 °C and humidity 50–60%) with free access to water and feed pellets. All procedures used in this study were approved by the ethics committees at the Animal Care and Use Committee, Kyushu University.

### 4.6. NAFL Model

All mice were anaesthetized with medetomidine (0.3 mg/kg), midazolam (4 mg/kg) and butorphanol (5 mg/kg) via i.p. injection, then osmotic pumps (ALZET, Cupertino, CA, USA) were intraperitoneally implanted for sustained administration of cilnidipine, 1,4-DHP or vehicle. In ob/ob mice, cilnidipine (5 mg/kg/day) was first administered for 3 weeks, then the concentration was increased to cilnidipine (20 mg/kg/day) for 3 weeks. HFD (D12492, Research Diets, Inc., New Brunswick, NJ, USA) was fed for 4 weeks prior to injection of vehicle or cilnidipine (5 mg/kg/day) to create HFD-induced diabetic mice. These mice were fed HFD for 14 weeks after administration. Ob/ob mice were fed HFD to create a model of severe NAFL. In ob/ob mice fed HFD, cilnidipine (5 mg/kg/day), 1,4-DHP (5 mg/kg/day) or vehicle was administered for 3 weeks. The liver was taken. Blood samples were collected from the caudal vena cava and centrifuged at 10,000× *g* for 10 min.

### 4.7. Plasma Biochemical Analysis

Plasma levels of AST, ALT, TG and TCHO were measured by using an automated biochemical analyzer (Fuji Dri-chem NX5000; Fujifilm Medical, Tokyo, Japan).

### 4.8. Transmission Electron Microscopy

Mouse liver tissues were pre-fixed for 3 h on ice using 2% paraformaldehyde solution containing 0.15 M sodium cacodylate and 2 mM CaCl_2_ (pH 7.4), and cut out into 1–2 mm cubes. After washing with 0.15 M cacodylate solution, block tissues were immersed in solution containing 2% osmium tetroxide, 1.5% potassium ferrocyanide, 0.15 M sodium cacodylate and 2 mM CaCl_2_ (pH 7.4) for 1.5 h at room temperature. After washing with distilled water, tissue cubes were immersed in 0.01 mg/mL thiocarbohydrazide solution for 40 min and then post-fixed using 2% osmium for 1 h. En bloc staining was performed by immersing tissues in a solution of 1% uranium acetate overnight at 4 °C and they were then immersed in an aqueous solution of lead aspartic acid for 60 min with oven-frying. After dehydration with graded series of ethanol and acetone, specimens were embedded with durcupan resin. The surface (70-nm thickness) of resin-embedded tissue was exposed using a diamond knife on an Ultracut UC7 (Leica Microsystems, Vienna, Austria). The surface of embedded tissue was imaged with a Veleta CCD camera (Olympus, Hamburg, Germany) equipped on JEOL1010 (JEOL, Tokyo, Japan). The area of LDs and mitochondria was analyzed using Image J software (Version 1.54g).

### 4.9. siRNA Transfection and Evaluation of the Effect of siRNA on LD Accumulation

HepG2 cells were transfected with 120 nM Drp1 or FLNA siRNA using Lipofectamine RNAiMAX reagent (Thermo Fisher Scientific) for 72 h. Stealth siRNAs for rat FLNa (#1, RSS308836; #2, RSS300837) and rat Drp1 (#1, RSS300121; #2, RSS300122) were obtained from Invitrogen. For live cell imaging, after transfection with 30 nM Drp1 or FLNA siRNA for 72 h, cells were treated with 30 μM BSA or PA for 24 h. Then cells were stained with Lipi-blue (200 nM) and Mitobright LT Green (100 nM) (Dojindo) and incubated at 37 °C. After staining with Lipi-blue and Mitobright LT Green, cells were washed with HBSS twice. LD images were captured using a confocal microscope (LSM900, ZEISS).

### 4.10. FAO Assay

The FAO efficacy was assessed using an XFp Extracellular Flux Analyzer (Agilent Technlogies, North Billerica, MA, USA). HepG2 cells were seeded onto the plates with a density of 1.0 × 10^4^ cells/well in DMEM. The cells were treated with or without cilnidipine (1 μM) for 1 h and then PA (200 μM) in substrate-limited medium (1 mM GlutaMax, 1% FBS, 0.5 mM L-carnitine, 0.5 mM Glucose in DMEM (Thermo Fisher Scientific, #A14430-01)) for 24 h. PA was BSA conjugated PA contained in Palmitate Oxidation Stress Test Kit (Agilent Technologies). BSA solution in the same kit was used as a control. Cells were incubated for 45 min in FAO assay medium (111 mM NaCl, 4.7 mM KCl, 1.25 mM CaCl_2_, 2.0 mM MgSO_4_, 1.2 mM NaH_2_PO_4_) supplemented with 5 mM HEPES, 0.5 mM L-carnitine, 2.5 mM glucose (pH 7.4). XF Palmitate-BSA FAO substrate (final concentration: 28.3 μM BSA control or 166.7 μM Palmitate conjugated to 28.3 μM BSA in 25 mM NaCl, pH7.2) was added just before assay. Oxygen consumption rate (OCR) was measured via the Mito stress test. Cellular bioenergetics were measured via automatic injections with 1.5 μM oligomycin, 2 μM carbonyl cyanide-p-(trifluoromethoxy) phenylhydrazone (FCCP), 1 μM rotenone and 1 μM antimycin A, in order. After OCR measurement, cells were fixed in 4% PFA and washed twice with PBS. After 30 min staining with DAPI, cell images were captured, and the number of cell nuclei was counted using a BZ-X800 microscope (KEYENCE). All values for OCR were normalized to the number of cells present in each well.

### 4.11. Real-Time RT-PCR

Total RNA was isolated from frozen mouse liver using RNeasy Fibrous Tissue Mini Kit (QIAGEN, Venlo, The Netherland) according to the manufacturer’s instructions. Complementary DNA (cDNA) was synthesized with Prime Script RT (Takara Bio, Siga, Japan). Real-time PCR was performed using Power SYBR Green PCR Master mix (Thermo Fisher Scientific). The primers are described in Supplementary Table S1.

### 4.12. Statistics

All results were expressed as mean ± SEM from at least 3 independent experiments and were considered significant when *p* < 0.05. We performed statistical analysis by using GraphPad Prism 9.0 (GraphPad Software, La Jolla, CA, USA). Statistical comparisons were determined using one-way or two-way ANOVA with Tukey’s post hoc test (for 3 or more groups).

### 4.13. Limitation of the Study

Our group lacks the technique to culture primary mouse hepatocytes. Therefore, we utilized human hepatocellular carcinoma cell line HepG2 as substitutes. Given the resemblance of mitochondrial morphology under hyperglycemic conditions to that of mouse liver tissues, we inferred that the cellular models partially mimic mitochondrial dysfunction in mouse liver. We also lack biochemical techniques and tools to assess mitochondrial biogenesis and mitophagy, and ER stress. Therefore, we only measured the mRNA expression profiles for mitochondrial fission/fusion-related factors and related factors for ER stress, oxidative stress, inflammation, mitophagy and mitochondrial quality control.

## Data Availability

The raw data supporting the conclusions of this article will be made available by the authors on request.

## Supplementary Materials

The following supporting information can be downloaded at: https://www.mdpi.com/article/10.3390/ijms25105446/s1.
