# Supplementary material for: Inhibition of Drp1–Filamin Protein Complex Prevents Hepatic Lipid Droplet Accumulation by Increasing Mitochondria–Lipid Droplet Contact
Source: Int J Mol Sci. 2024 May 17;25(10):5446. doi: 10.3390/ijms25105446 (PMC11122359; doi:10.3390/ijms25105446)
Supplement: Supplementary file 1 [file ijms-25-05446-s001.zip › ijms-2987773-supplementary.pdf]

International Journal of
Molecular Sciences

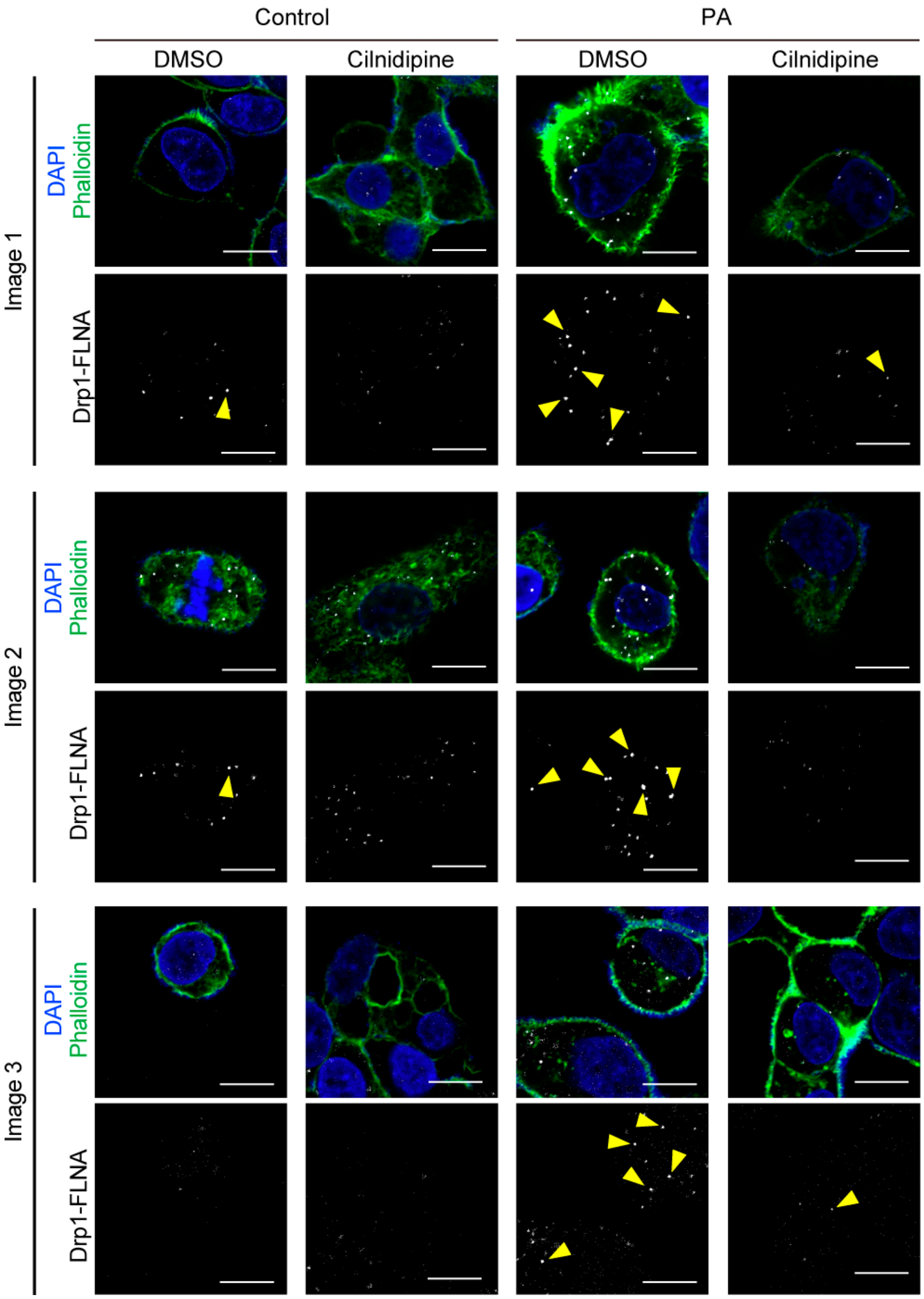

Supplemental Figure S1. Effect of cilnidipine on the PA-induced Drp1-FLNA complex formation in HepG2. Supplemental images of PLA between Drp1 and FLNA. PLA signals are shown as white spots (yellow arrowhead) counterstained with phalloidin (green) and DAPI (blue). HepG2 cells were treated with 30 µM of PA with or without cilnidipine. Scale bars: 10 µm.

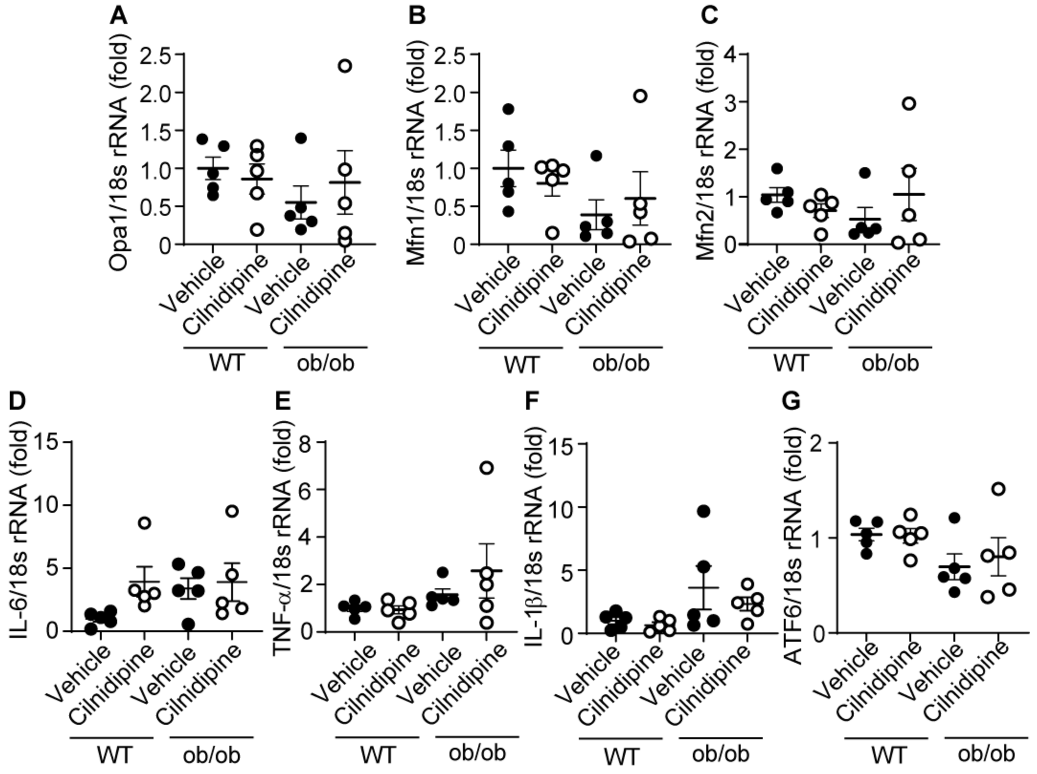

Supplemental Figure S2. Changes in mRNA expression levels related to mitochondria fusion, inflammation, and ER stress in ob/ob mice. (A-C) Gene expression of mitochondrial fusion-related proteins. The expression of Opa1 (A), Mfn1 (B), and Mfn2 (C). (D-F) Gene expression of inflammation-related proteins. The expression of IL-6 (D), TNF-α (E), and IL-1β (F). (G) The expression level of ATF6 gene. Data are means ± SEM (n=5 mice in each group). Significance was determined using one-way ANOVA followed by Tukey's comparison test.

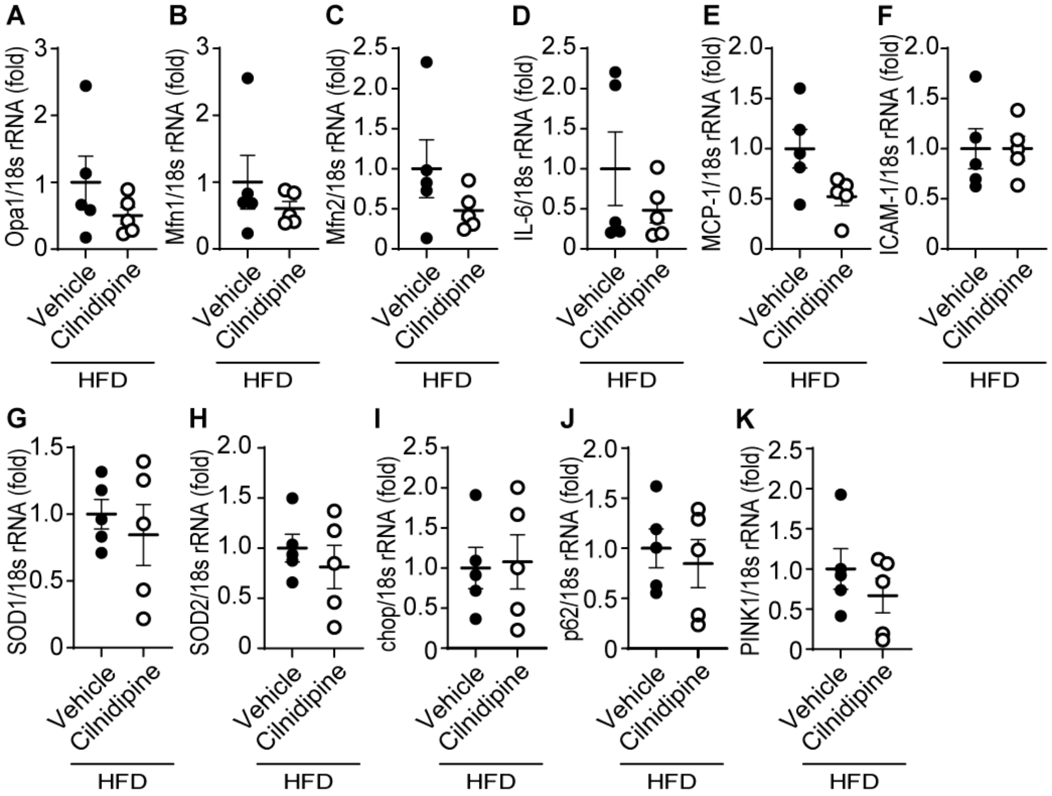

Supplemental Figure S3. Changes in mRNA expression levels related to mitochondria fusion, inflammation, and ER stress in WT mice fed HFD. (A-C) Gene expression of mitochondrial fusion-related proteins. The expression of Opa1 (A), Mfn1 (B), and Mfn2 (C). (D-F) Gene expression of inflammation-related proteins. The expression of IL-6 (D), MCP-1 (E), and ICAM-1 (F). (G, H) Gene expression of ROS-related proteins. The expression of SOD1 (G), and SOD2 (H). (I, J) Gene expression of ER stress-related proteins. The expression of chop (I), and p62 (J). (K) mRNA expression of PINK1. Data are means ± SEM (n=5 mice in each group). Significance was determined using one-way ANOVA followed by Tukey's comparison test.

Ha

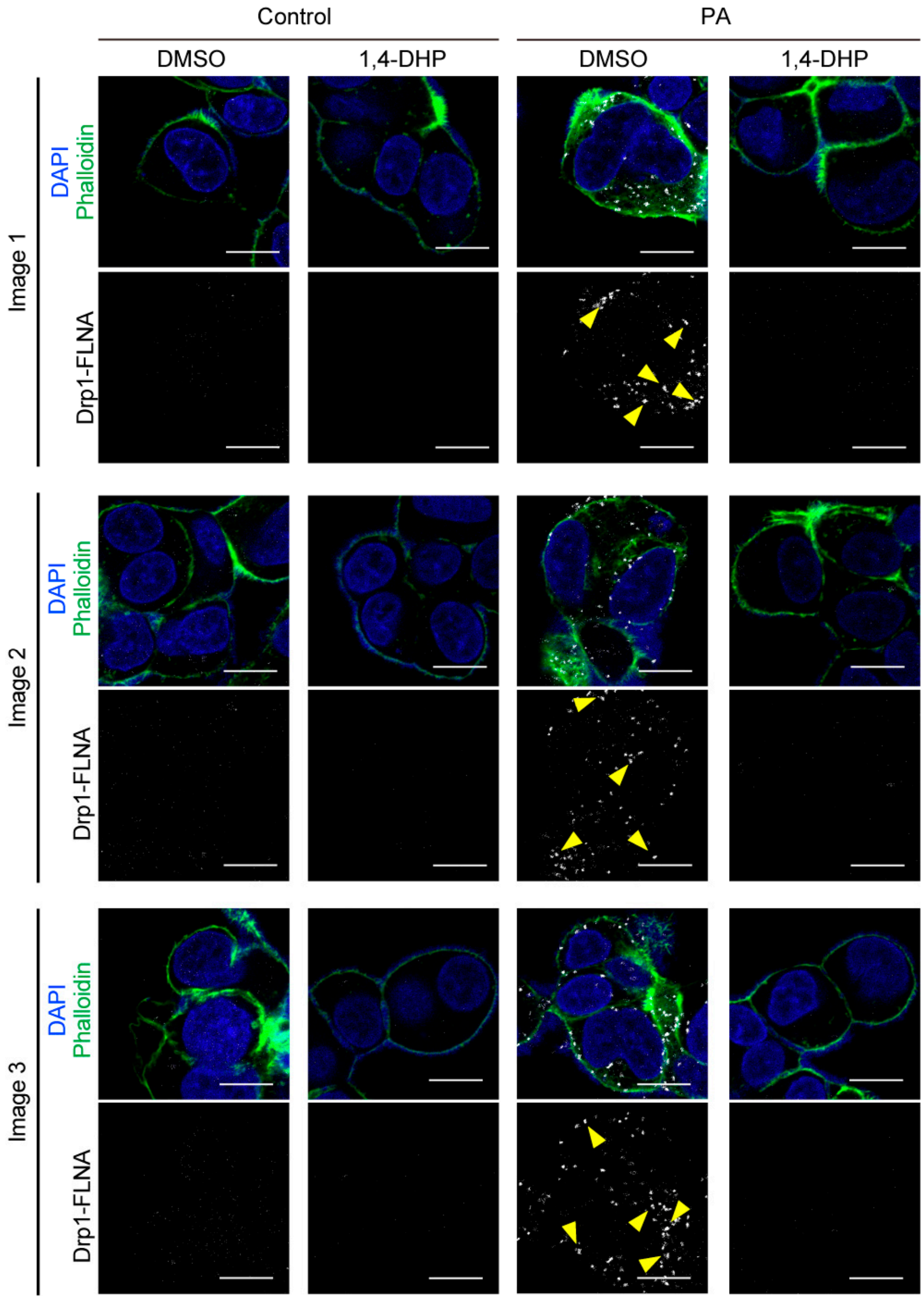

Supplemental Figure S4. Effect of 1,4-DHP on the PA-induced Drp1-FLNA complex formation in HepG2. Representative images of PLA between Drp1 and FLNA. PLA signals are shown as white spots (yellow arrowhead) counterstained with phalloidin (green) and DAPI (blue). HepG2 cells were treated with 30 µM of PA with or without 1,4-DHP. Scale bars: 10 µm.

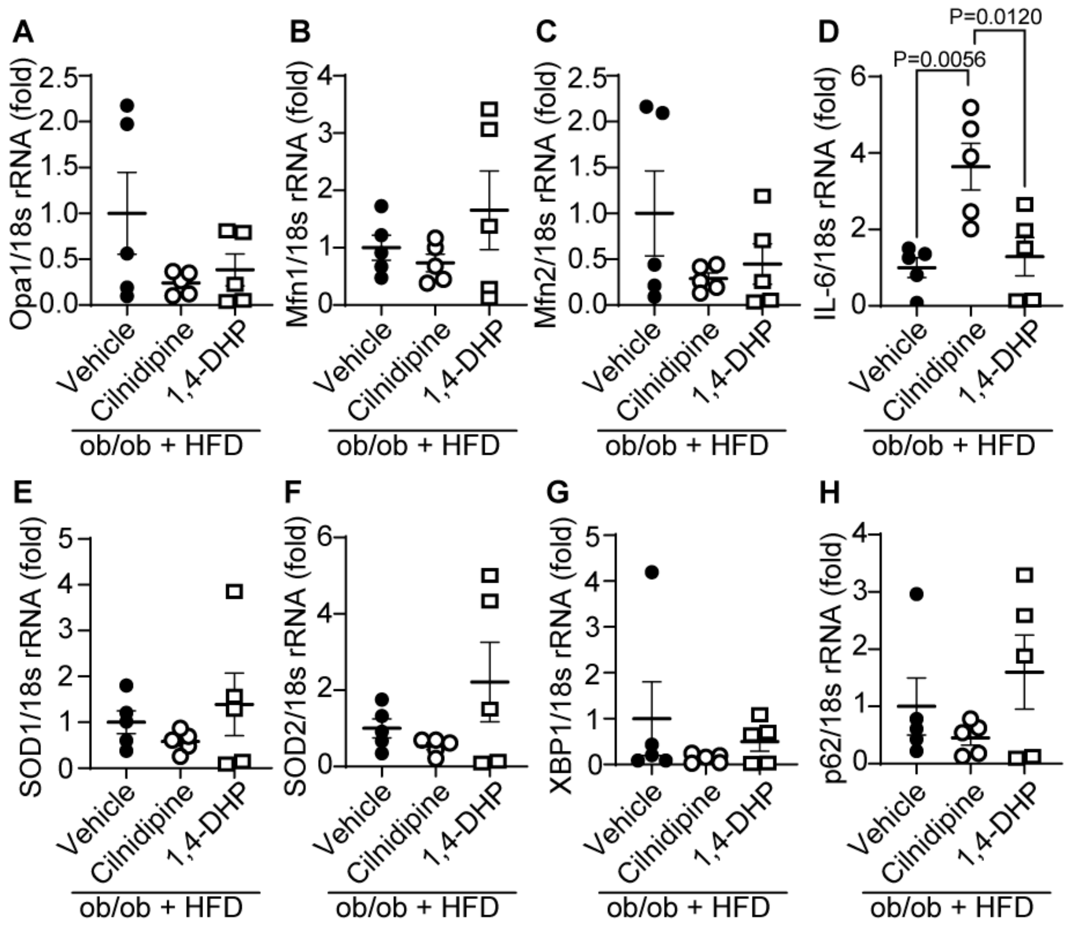

Supplemental Figure S5. Changes in mRNA expression levels related to mitochondria fusion, inflammation, and ER stress in ob/ob mice fed HFD. (A-C) Gene expression of mitochondrial fusion-related proteins. The expression of Opa1 (A), Mfn1 (B), and Mfn2 (C). (D) The expression of IL-6. (E, F) Gene expression of ROS. The expression of SOD1 (E), and SOD2 (F). (G, H) Gene expression of ER stress. The expression of XBP1 (G), and p62 (H). Data are means ± SEM (n=5 mice in each group). Significance was determined using one-way ANOVA followed by Tukey's comparison test.

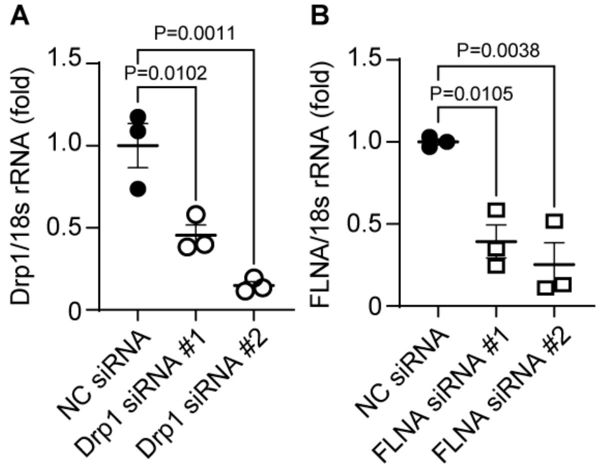

Supplemental Figure S6. Knockdown efficiencies of siRNAs for Drp1 and FLNA in HepG2. (A, B) mRNA expression levels of Drp1 (A), and FLNA (B) in HepG2 cells. Data are means ± SEM (n=3 in each group). Significance was determined using two-way ANOVA followed by Tukey's comparison test.

Supplementary Table S1 Primer list

No.	Gene		Primer Sequence (5' - 3')
1	Drp1	Forward	GATGCCATAGTTGAAGTGGTGAC
		Reverse	CCACAAGCATCAGCAAAGTCTGG
2	FLNA	Forward	CTTATCGCGCTGTTGGAGGT
		Reverse	GCCACCGACACGTTCTCAA
3	Opa1	Forward	TGGAAAATGGTTCGAGAGTCAG
		Reverse	CATTCCGTCTCTAGGTTAAAGCG
4	Mfn1	Forward	ATGGCAGAAACGGTATCTCCA
		Reverse	CTCGGATGCTATTCGATCAAGTT
5	Mfn2	Forward	GTGGAATACGCCAGTGAGAAGC
		Reverse	CAACTTGCTGGCACAGATGAGC
6	IL-6	Forward	AAGGGCCAGGGATCTGTAAG
		Reverse	TCTCTTGTTGCTCCCCAAAG
7	TNF-alpha	Forward	ATGAGCACAGAAAGCATGATCCGC
		Reverse	CCAAAGTAGACCTGCCCGGACTC
8	IL-1 beta	Forward	ATGGCAACTGTTCCTGAACTCAACT
		Reverse	CAGGACAGGTATAGATTCTTTCCTTT
9	MCP-1	Forward	TTAAAAACCTGGATCGGAACCAA
		Reverse	GCATTAGCTTCAGATTTACGGGT
10	ICAM1	Forward	GTGTGCCATGCCTTTAGCTC
		Reverse	CTGATCTTTCTCTGGCGGTT
11	SOD1	Forward	AACCAGTTGTGTTGTCAGGAC
		Reverse	CCACCATGTTTCTTAGAGTGAGG
12	SOD2	Forward	CAGACCTGCCTTACGACTATGG
		Reverse	CTCGGTGGCGTTGAGATTGTT
13	ATF6	Forward	TCGCCTTTTAGTCCGGTTCTT
		Reverse	GGCTCCATAGGTCTGACTCC
14	XBP1	Forward	CTGAGTCCGAATCAGGTGCAG
		Reverse	GTCCATGGGAAGATGTTCTGG
15	p62	Forward	GCTGCCCTATACCCACATCT
		Reverse	CGCCTTCATCCGAGAAAC
16	chop	Forward	CACCACACCTGAAAGCAGAA
		Reverse	CGTTTCCTGGGGATGAGATA
17	PINK1	Forward	CTTATAGGAAAGGGCCCGGATGTCG
		Reverse	GATGATGTTAGGGTGTGGGGCAAGC

Ha

18	18srRNA	Forward	ATTAATCAAGAACGAAAGTCGCAGGT
		Reverse	TTTAAGTTTCAGCTTTGCAACCATACT
19	Human 18srRNA	Forward	CTACCACATCCAAGGAAGCA
		Reverse	TTTTTCGTCACTACCTCCCCG

Ha
